# Anson B. Hayden

**Published:** 1842-09

**Authors:** 


Died at Savannah, Ga. of pulmonary consumption, on the 29th of June last, in the 52d year of his age, Anson B. Hayden, Dental Surgeon, formerly of Windsor, Conn. He commenced his professional studies with his brother, H. H. Hayden, M. D., of Baltimore, in 1818, and continued through '18 and '19, attending at the same time most of the lectures of the University of Maryland. After having finished his professional education, he commenced practice in Savannah, where he resided for several years; finding, however, the climate to disagree with his health, he removed to Washington City, where he remained in successful practice, until attacked with his last illness, which obliged him to seek a warmer climate, where he died.

This we believe, is the first time it has become our painful duty to announce the death of one of our members since the organization of the American Society of Dental Surgeons. At the last meeting of the society, suitable resolutions were passed, expressive of their sense of the loss they had sustained, and directing the usual badges of mourning on such occasions; also of condolence with the family and friends of the deceased, with whom we most sincerely sympathize, and pray that they may have abundantly the consolation which religion alone can afford. M.

